# Rare and uncommon tumours of the female pelvis: what the radiologist should know

**DOI:** 10.1093/bjr/tqaf176

**Published:** 2025-07-24

**Authors:** Silvia Bottazzi, Luca Russo, Veronica Celli, Anna Rame, Alessandra Iacono, Guido Imbemba, Evis Sala, Benedetta Gui

**Affiliations:** Dipartimento di Diagnostica per Immagini e Radioterapia Oncologica, Fondazione Policlinico Universitario Agostino Gemelli IRCCS, Rome 00168, Italy; Dipartimento Universitario di Scienze Radiologiche ed Ematologiche, Università Cattolica del Sacro Cuore, Rome 00168, Italy; Dipartimento di Diagnostica per Immagini e Radioterapia Oncologica, Fondazione Policlinico Universitario Agostino Gemelli IRCCS, Rome 00168, Italy; Dipartimento Universitario di Scienze Radiologiche ed Ematologiche, Università Cattolica del Sacro Cuore, Rome 00168, Italy; Dipartimento di Diagnostica per Immagini e Radioterapia Oncologica, Fondazione Policlinico Universitario Agostino Gemelli IRCCS, Rome 00168, Italy; Dipartimento Universitario di Scienze Radiologiche ed Ematologiche, Università Cattolica del Sacro Cuore, Rome 00168, Italy; Dipartimento Universitario di Scienze Radiologiche ed Ematologiche, Università Cattolica del Sacro Cuore, Rome 00168, Italy; Dipartimento Universitario di Scienze Radiologiche ed Ematologiche, Università Cattolica del Sacro Cuore, Rome 00168, Italy; Dipartimento di Diagnostica per Immagini e Radioterapia Oncologica, Fondazione Policlinico Universitario Agostino Gemelli IRCCS, Rome 00168, Italy; Dipartimento Universitario di Scienze Radiologiche ed Ematologiche, Università Cattolica del Sacro Cuore, Rome 00168, Italy; Dipartimento di Diagnostica per Immagini e Radioterapia Oncologica, Fondazione Policlinico Universitario Agostino Gemelli IRCCS, Rome 00168, Italy

**Keywords:** magnetic resonance imaging, female genital neoplasms, rare tumours

## Abstract

Gynaecological tumours present a broad spectrum of histological subtypes due to the diverse anatomical and tissue origin of the reproductive organs. Rare tumours affect less than 6 per 100 000 individuals annually, posing significant challenges in diagnosis and management due to limited clinical awareness. Indeed, treatment protocols rely on options developed for more common histotypes, which may have limited efficacy on these rare tumours. In recent years, collaborative international efforts have started to address these gaps, improving standards of care. A comprehensive understanding of rare tumours’ clinical and imaging features is necessary for radiologists in order to provide clinicians with useful information for treatment planning. In this review, we adopted an organ-based outline, describing rare tumours of the uterine corpus (leiomyosarcoma, endometrial stromal sarcoma, carcinosarcoma), cervix (gastric-type adenocarcinoma), and ovary (cystadenofibroma, lipid-poor teratoma, struma ovarii, immature teratoma, dysgerminoma). Additionally, tumours occurring at multiple sites, including lymphoma, neuroendocrine tumours, aggressive angiomyxoma and metastases are discussed. The objective is to help radiologists become familiar with these uncommon entities, ultimately increasing awareness on this topic.

## Introduction

Gynaecological tumours can arise from any structure within the genital tract. The genital tract’s anatomical complexity, with its multiple organs and diverse tissue types, predisposes for a wide variety of potential tumour subtypes. However, many of these histological subtypes are exceedingly rare, and most radiologists may never encounter them in their careers.^1–3^

Rare tumours affect less than 6 per 100 000 individuals a year and pose significant diagnostic and management challenges due to limited awareness of their imaging characteristics, clinical behaviour, and optimal treatment approaches, as these patients are not routinely involved in clinical trials.^4–6^

Consequently, patients with rare tumours are frequently misdiagnosed or mistreated due to the absence of specific guidelines, with management defaulting to strategies designed for more common histological subtypes.^6^ Additionally, evolving tumour classifications add complexity to understanding these diseases, eventually confusing clinicians and radiologists.^2^^,^^7^^,^^8^

Encouragingly, increased interest in this topic has led to several international collaborations aimed at filling the existing gaps and, consequently, improving the quality of care for these patients.^9–12^

In many cases, rare tumours are diagnosed through biopsy. Therefore, radiologists must understand the tumour’s behaviour, aggressiveness, and patterns of spread rather than solely focusing on lesion characterisation.

This review aims to explore the typical radiological features and behaviour of uncommon gynaecological tumours to help radiologists assess these entities, emphasising what is clinically relevant for patient management. Although we primarily focused on malignant tumours, we also included certain benign lesions that often mimic malignancy and may lead to misdiagnosis and overtreatment—such as cystadenofibroma, lipid-poor teratoma, and struma ovarii—or present considerable diagnostic and therapeutic difficulties due to their local invasiveness, as in the case of aggressive angiomyxoma.

## Uterine body tumours

Rare uterine body tumours are predominantly of mesenchymal origin. The most common type is uterine sarcoma, accounting for 3%-9% of uterine neoplasms. Uterine sarcomas most commonly arise from the outer myometrium, as in leiomyosarcomas; however, they may occasionally develop from the sparse mesenchymal cells within the endometrial layer, such as in endometrial stromal sarcomas.^8^^,^^13^

In contrast, carcinosarcomas are mixed tumours containing both mesenchymal and epithelial components. They have been recently reclassified as a rare and aggressive subtype of endometrial cancer, reflecting their primarily epithelial origin.^7^

### Leiomyosarcoma

**Figure 1.**
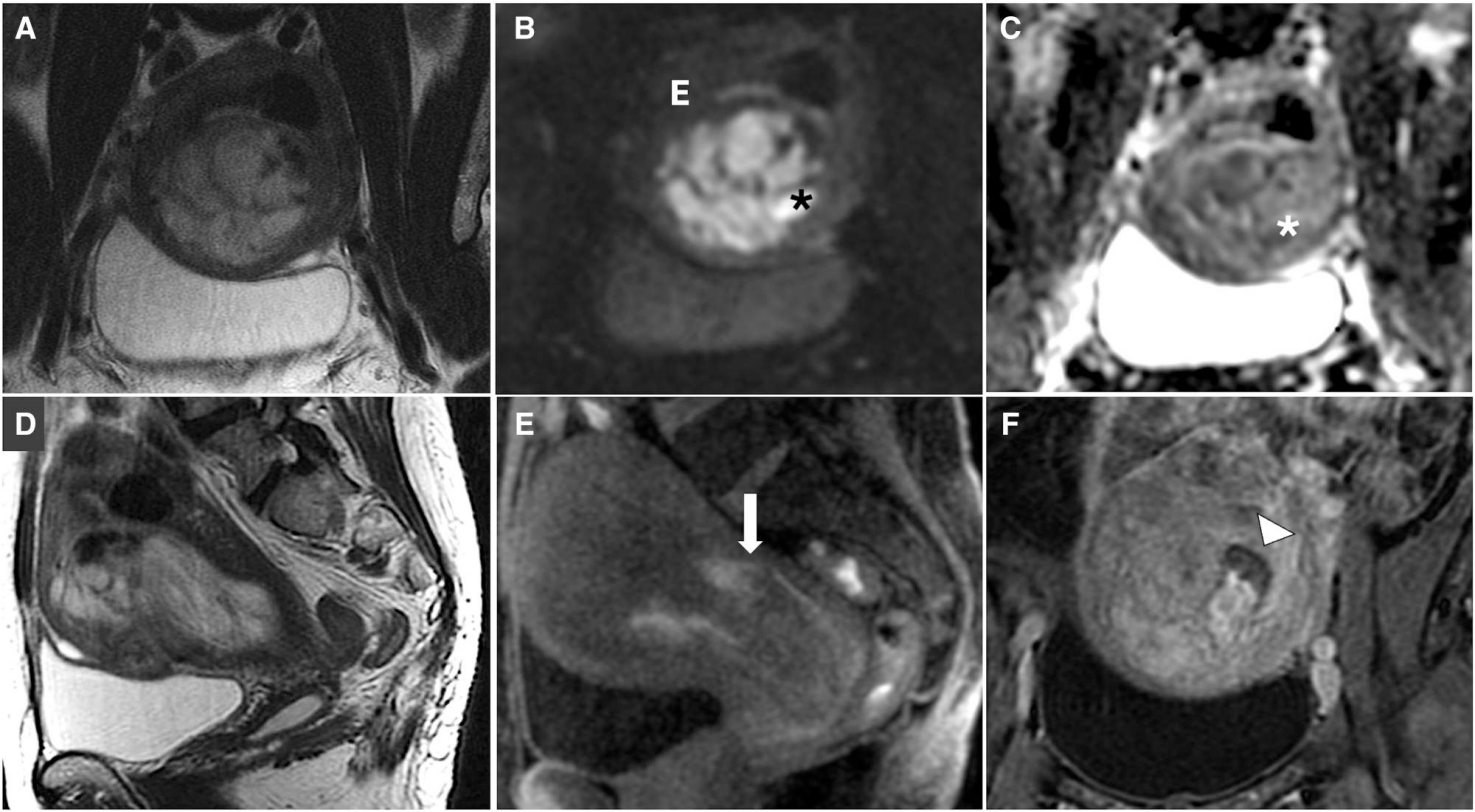
Leiomyosarcoma. MR images show a large, irregular lesion with high SI on T2-WI (A, D), with areas of viable tissue (asterisk) demonstrating higher SI on DWI (B) compared with the endometrium (E) and corresponding low SI on ADC map (C). Heterogeneous appearance on T1-WI (E) and postcontrast images (F) is due to the presence of haemorrhage (arrow) and necrosis (arrowhead). Abbreviations: SI = signal intensity; WI = weighted imaging.

Leiomyosarcoma (LMS) is the most common uterine sarcoma, accounting for 41%-60% of cases, followed by endometrial stromal sarcoma and adenosarcoma.^14–16^ The median age at diagnosis is 60 years; thus, a rapidly growing mass in a postmenopausal woman not on hormonal treatment should raise the suspicion of a LMS.^15^^,^^17^^,^^18^ Of note, only 0.2% of LMS arise from a preexisting leiomyoma.^19^^,^^20^ Nodal and peritoneal involvement are common, with metastases to the lungs, peritoneum, or upper abdomen present in up to 33% of cases at diagnosis.^15^^,^^20^ MRI demonstrated high accuracy (97.6%) in distinguishing LMS from leiomyomas.^14^ In recent years, different scoring systems have been proposed based on size, borders, and MRI-signal characteristics to aid the differential diagnosis. On MRI, LMS typically appear as large, irregular lesions, showing intermediate-to-high signal intensity (SI) on T2-weighted imaging (WI). They often exhibit heterogeneous contrast enhancement due to haemorrhage and necrosis.^15–17^^,^^20^ Quantification of necrotic areas, which are generally ≤20% in leiomyomas compared with LMS, may improve diagnostic specificity.^17^ Also, diffusion restriction with diffusion weighted imaging (DWI) SI equal to or higher than that of the endometrium (or lymph nodes) suggests LMS (Figure 1).

Quantification of apparent diffusion coefficient (ADC) values is recommended, with proposed cut-offs ranging from 0.79 to 1.23 × 10^−^³ mm^2^/s. An ADC of 0.905 × 10^−^³ mm^2^/s or lower indicates malignancy.^15^^,^^20^^,^^21^ To ensure accuracy, ADC values should be measured in viable tissue, avoiding haemorrhagic regions which can exhibit low ADC values.^15^

### Endometrial stromal sarcoma

Endometrial stromal sarcoma (ESS), a subset of endometrial stromal tumours, is classified as low-grade or high-grade.^22^ ESS typically affects younger women compared with endometrial carcinoma, particularly in cases of low-grade ESS.^20^^,^^23^

On MRI, ESS appears as a large, endometrial-based lesion with significant myometrial involvement.^24^ A distinguishing feature is the presence of hypointense bands on T2-WI, representing preserved myometrium within areas of myometrial involvement. These bands create a “worm-like appearance” that aids differentiation from leiomyomas.^24–27^ The lesion may also contain cystic areas.^24^

**Figure 2.**
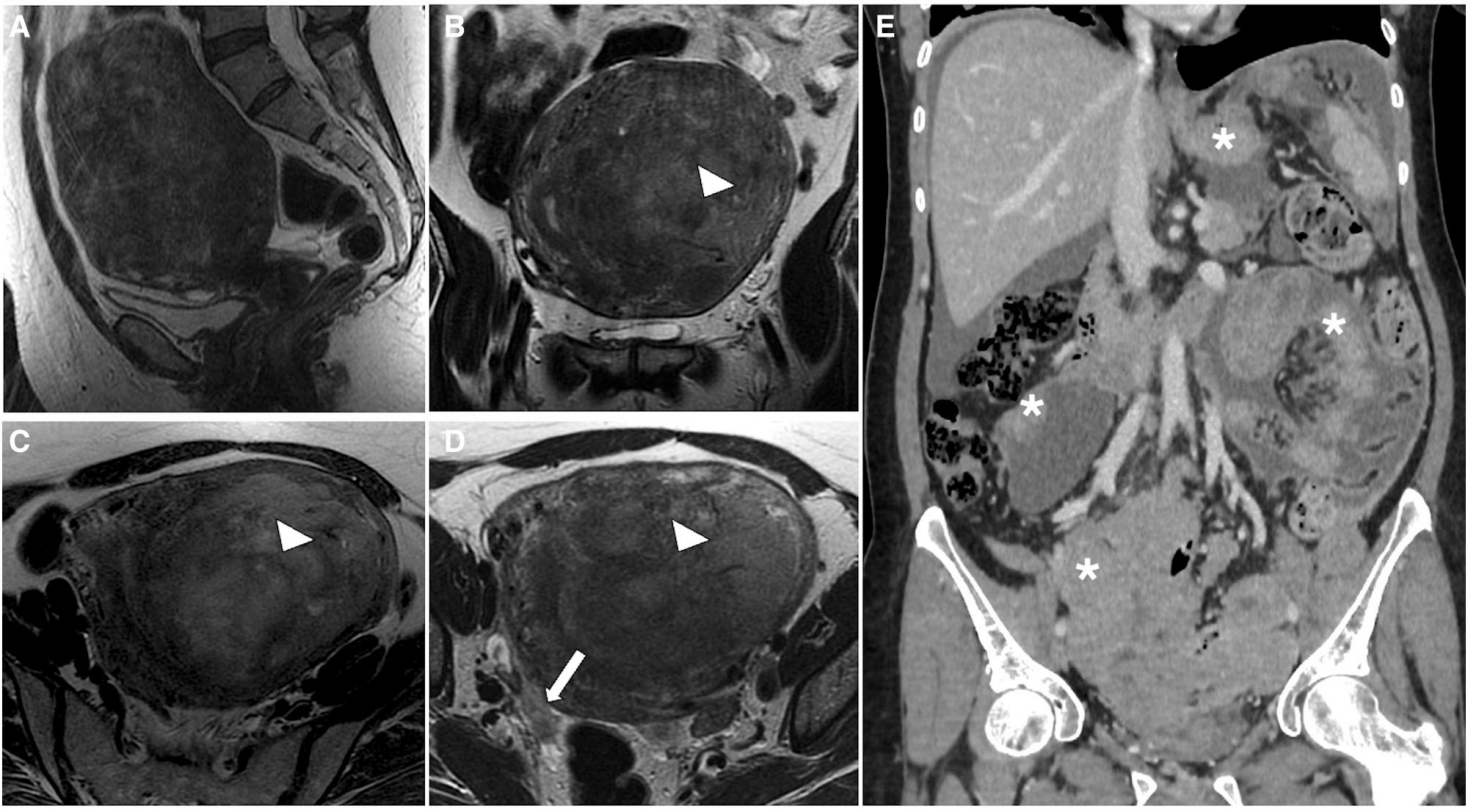
High-grade endometrial stromal sarcoma. Sagittal (A) and axial T2-WI (B-D) show the typical appearance of a high-grade ESS with hypointense bands of preserved myometrium within areas of myometrial involvement (arrowheads). The tumour extends through the right fallopian tube (arrow) into the pelvic peritoneum (D). The aggressive nature of the tumour is demonstrated by the diffuse peritoneal involvement seen on CT (asterisks in E). Abbreviations: ESS = endometrial stromal sarcoma; WI = weighted imaging.

ESS typically shows heterogeneous enhancement on MRI, sometimes with a feather-like pattern, appearing iso- or hyperintense relative to normal myometrium.^27^^,^^28^ Typical findings include intramyometrial nodules, intravascular extension, and continuous spread to surrounding structures via the fallopian tube or uterine ligaments^19^^,^^24^^,^^27^^,^^29^ (Figure 2). However, MRI is primarily used for staging after an endometrial biopsy rather than preoperative diagnosis.^26^ High-grade ESS demonstrates more aggressive and destructive behaviour compared with low-grade ESS.^23^

### Carcinosarcoma

**Figure 3.**
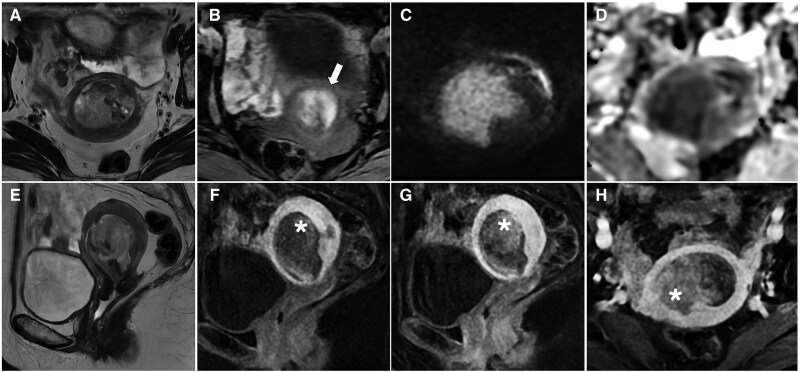
Endometrial carcinosarcoma. MR images show a large endometrial-based lesion extending through the internal os, demonstrating heterogeneous appearance on T2-WI (A, E) and T1-WI (B) due to the presence of haemorrhagic areas (arrow), and marked impeded diffusion (C, D). Arterial (F), venous (G), and delayed (H) postcontrast images show progressive and persistent enhancement (asterisk).

**Figure 4.**
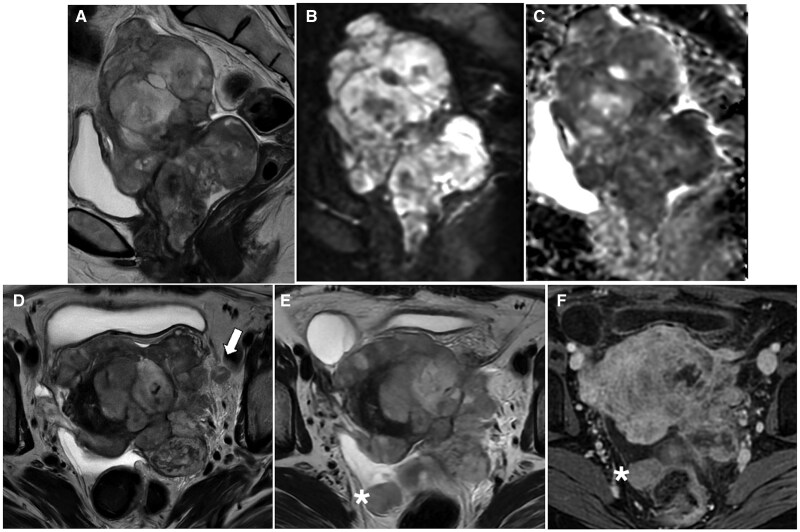
Cervical carcinosarcoma. MRI shows a large lesion replacing the normal uterine anatomy, with heterogeneous appearance on T2-WI (A, D, E) and marked impeded diffusion (B, C). There is also evidence of nodal (arrow in D) and peritoneal metastases (asterisks in E and F), reflecting the aggressive nature of carcinosarcoma.

Carcinosarcoma, formerly known as malignant mixed Mullerian tumour, is the rarest subtype of endometrial cancer and an extremely uncommon occurrence in the cervix.^30^ Once classified as a uterine sarcoma, it represents less than 1% of uterine malignancies but accounts for approximately 15% of related deaths due to its aggressive nature.^15^^,^^31^^,^^32^ It exhibits a high propensity for lymphatic spread, peritoneal dissemination, and distant metastases, with nodal involvement at diagnosis in up to 35% of cases and metastatic disease in over 10%.^32^ On MRI, carcinosarcomas typically appear as large solid masses within the endometrial cavity, often extending through the cervical os, with frequent myometrial or cervical invasion. They demonstrate inhomogeneously hyperintense SI on T2-WI due to haemorrhage and necrosis.^19^^,^^23^ Persistent, progressive enhancement may help distinguish them from other subtype of endometrial cancer^26^^,^^30^ (Figures 3 and 4).

## Cervical tumours

Cervical cancer is the fourth most common neoplasm in women.^33^ Squamous cell carcinoma (SCC) accounts for 80% of cases and is generally associated with human papillomavirus (HPV) infection. Conversely, adenocarcinomas (AC) are a more heterogeneous group, accounting for approximately 10%-20% of cervical cancer cases.^34^ The classification of AC has been recently revised, dividing AC into 2 main groups based on morphology and HPV association.^2^ HPV-associated AC represents approximately 85% of AC, with usual-type and mucinous-type AC being the most frequent subtype, while gastric-type AC is the main subtype of HPV-independent AC.^34^^,^^35^

### Gastric-type adenocarcinoma of the cervix

Gastric-type AC represents approximately 90% of HPV-independent AC, making up to 10% of all AC cases. Of note, the incidence of gastric-type AC is higher in patients with Peutz-Jeghers syndrome.^34^^,^^36^^,^^37^ Patients may present with watery vaginal discharge or abnormal bleeding.^36^ This subtype includes tumours with variable grades of differentiation and is also known as adenoma malignum, a well-differentiated form, or minimal deviation adenocarcinoma.^2^

Gastric-type AC typically arises in the upper third of the cervical canal but frequently extends to involve the entire cervix at diagnosis due to its aggressive nature. Unlike SCC, which typically presents as a mass-forming, solid lesion that replaces normal cervical tissue, gastric-type AC has a more insidious presentation, making it diagnostically challenging for radiologists. MRI features of gastric-type AC include infiltrative, endophytic growth centred within the cervical stroma, which often results in underestimation of the disease extent and a characteristic solid-cystic morphology with small cysts embedded within the solid component. When the solid component is small, diffusion restriction on DWI images may be minimal.^35^^,^^36^^,^^38^^,^^39^ In these cases, postcontrast T1-WI may help identifying the solid component.^36^

Histologic diagnosis can be challenging as deeply located tumour glands necessitate deep biopsies, and well-differentiated forms of gastric-type AC exhibit cytologic features that overlap with benign conditions on smear tests.^40^^,^^41^

**Figure 5.**
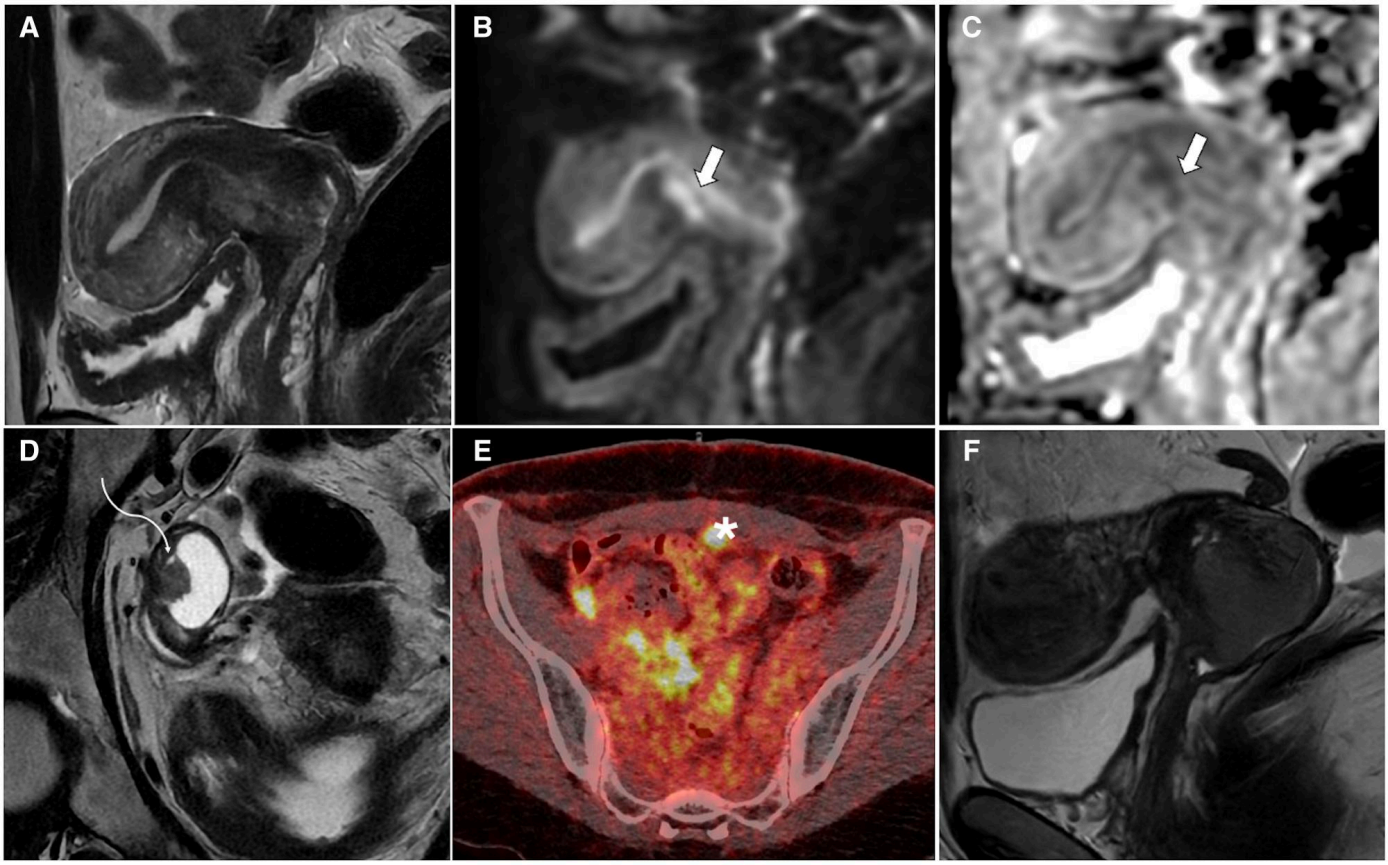
Gastric-type adenocarcinoma. Sagittal T2-WI (A) shows a subtle solid-cystic lesion with ill-defined margins and endophytic growth in the cervical stroma. This infiltrative pattern is typical of the gastric-type adenocarcinoma. Mild-to-moderate impeded diffusion is noted (B, C), leading to underestimation of the disease extent (arrow). Ovarian (curved arrow) and peritoneal (asterisk) involvement can also be noted (D, E). Conversely, SCC presents as a sharply demarcated mass-forming solid lesion on T2-WI (F). Abbreviations: SCC = squamous cell carcinoma.

These tumours often present at advanced stages and are associated with a higher incidence of local and distant metastases. The peritoneum and the adnexa are common sites of disease, with ovarian metastases found in up to 25% of cases. Hence, careful assessment of surrounding structures is required^42^^,^^43^ (Figure 5).

It can be challenging to differentiate gastric-type AC from other cystic cervical lesions, such as lobular glandular hyperplasia, Nabothian cysts, and cervical polyps. Unlike the subtle, infiltrative growth characteristic of gastric-type AC, benign lesions generally present with well-defined margins. Additionally, Nabothian cysts lack a solid component and are more superficially located, while cervical polyps demonstrate exophytic growth patterns, distinctly contrasting with the infiltrative behaviour of gastric-type AC.^35^^,^^36^

## Ovarian tumours

Most ovarian tumour subtypes are rare, and differential diagnosis on MRI can be challenging. Thus, MRI images should always be integrated with the patient’s age, clinical history and serum markers.^44^ In the following paragraphs, we will focus on those presenting specific features that can help the radiologist in their recognition. Some of these entities, such as cystadenofibroma and lipid poor teratoma, although predominantly benign, can mimic malignant epithelial tumours. However, they present with distinctive imaging characteristics that enable correct identification.

### Cystadenofibroma

Ovarian cystadenofibroma accounts for 1.7% of ovarian tumours and is classified as benign, borderline, or malignant based on the degree of epithelial proliferation, with the malignant form being extremely rare.^45^^,^^46^ For radiologists, it is crucial to recognise the cystadenofibroma MRI features, as it is a frequent mimicker of borderline and malignant lesions.^47^^,^^48^

On MRI, cystadenofibromas typically appear as uni- or multilocular lesions with smooth margins and a variable solid component. Typical findings include thickened septa and irregular borders, with mural nodules and papillary projections in up to 33% of cases.^45^^,^^49^ A key distinguishing feature is the solid component’s marked low-SI on T2-WI.^44^ This solid component usually exhibits no diffusion restriction and progressive enhancement, displaying a type I perfusion curve consistent with its fibrous nature.^47^^,^^49^ Although rare, borderline cystadenofibromas may demonstrate intermediate-risk or high-risk time-intensity curves on dynamic contrast-enhanced imaging, indicative of increased vascularity.^47^

**Figure 6.**
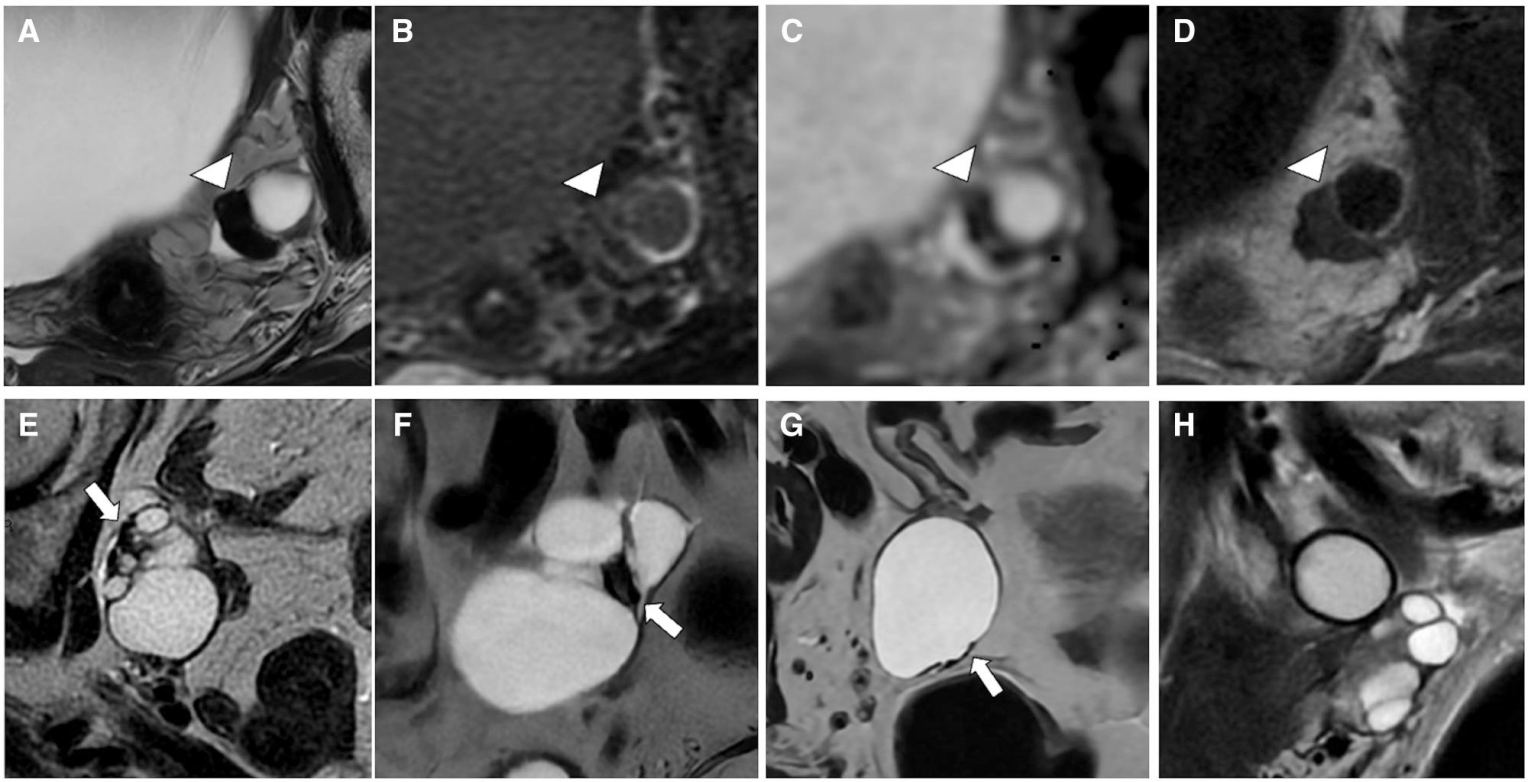
Cystadenofibroma. Pelvic MRI showing a multilocular cystic lesion with a nodular solid component (arrowhead) (A-D). The solid component has low SI on T2-WI (A) and no impeded diffusion (B, C). On post-contrast T1-WI (D), mild and late enhancement is seen. Three morphological patterns of cystadenofibroma have been described. Type I or “black sponge” (E, F): multilocular cystic lesion with fibrotic nodular solid component (very low SI on T2-WI) along the septa (arrow in E) or protruding into the loculi (F). Type II or “carpet-like” (G): unilocular cystic lesion with a plaque-like thickening of the cystic wall (arrow). Type III or “purely cystic” (H): no solid component can be noted in this pattern. Abbreviations: SI = signal intensity; WI = weighted imaging.

Three morphological patterns of cystadenofibroma have been described, ranked by frequency (Figure 6):

- Type I, “black-sponge”: multilocular cystic mass with fibrotic nodular components along the septa or protruding into the loculi.
- Type II “carpet-like”: uni or bilocular cystic mass with focal pseudo nodular or plaque-like thickening of the cyst walls.
- Type III “purely-cystic”: cystic mass without evidence of a solid component.

Notably, a contralateral lesion is found in approximately 50% of cases, most often representing benign lesions, particularly cystadenofibromas.^49^

### Lipid-poor teratoma, struma ovarii, and immature teratoma

Mature cystic teratoma (MCT) is the most common germ cell tumour, characterized by intralesional fat. However, in 15% of cases, fat is absent in the cystic component. Notably, only 6% of MCTs are entirely devoid of fat.

A small amount of fat may be present in the cystic wall or Rokitansky nodule, which, therefore require careful evaluation to avoid misdiagnosing lipid-poor MCT for other borderline or malignant epithelial lesions. In such cases, T1-WI in- and opposed-phase can aid in detecting subtle fat.^50–52^

Complications of MCT include torsion, rupture, and malignant transformation.^53^^,^^54^ Malignant transformation, although rare (1%-2%) and typically occurring in older patients, should be suspected if a large, enhancing solid component with transmural extension is observed, usually originating in the Rokitansky nodule. A typical Rokitansky nodule, in contrast, is usually smooth and rounded.^50^

**Figure 7.**
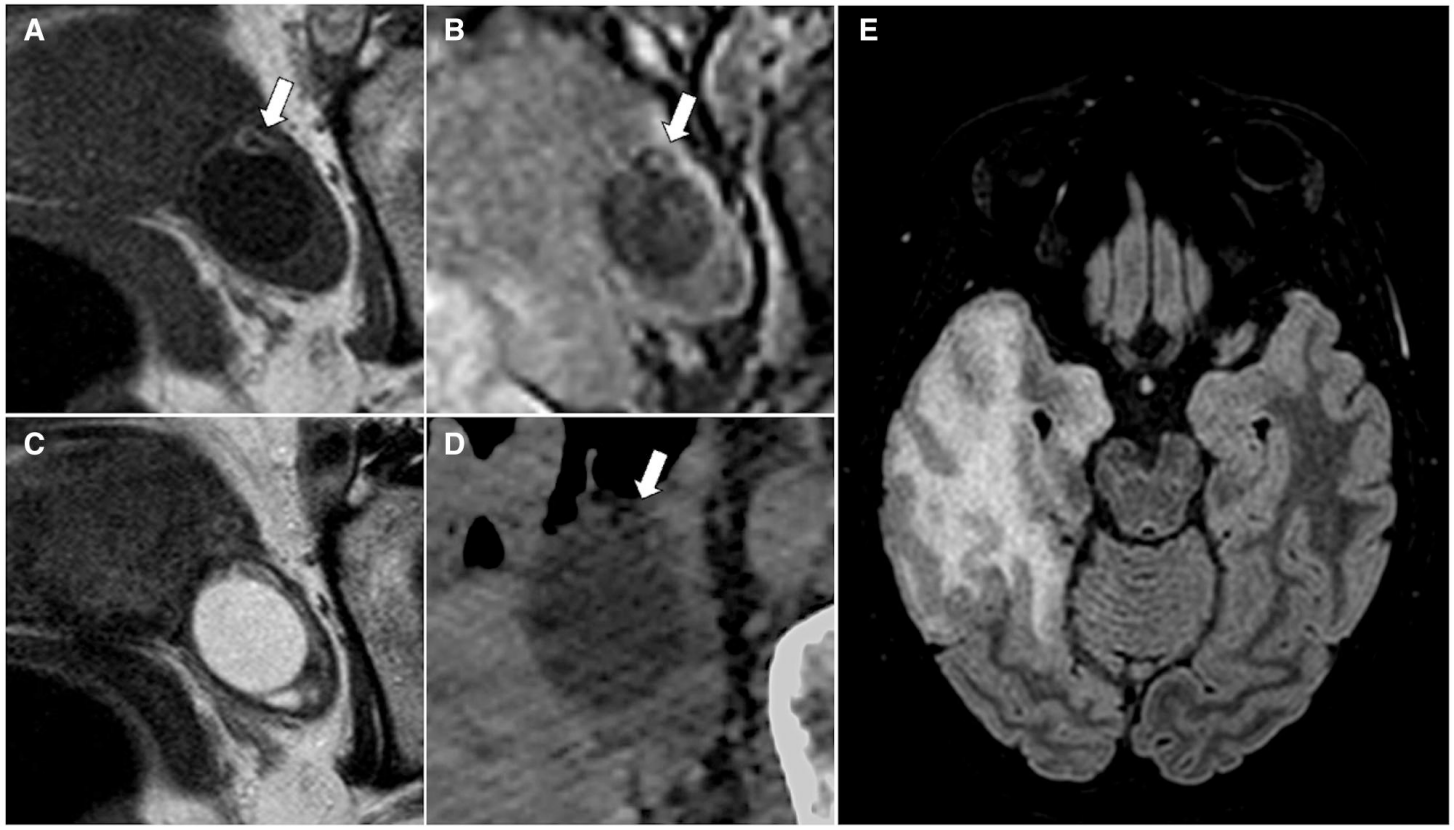
Lipid-poor mature cystic teratoma. Pelvic MRI (A-C) and CT (D) in a 30-year-old patient with a small left adnexal lesion. The fat component is confined to the cystic wall and shows high SI on T1-WI (arrow in A), low SI on T1-WI with fat saturation (B) and hypodense appearance on CT image (D). The patient underwent an MRI brain because presenting with confusion, amnesia, loss of consciousness and seizures. FLAIR sequence (E) shows oedematous changes in the right temporal lobe consistent with the diagnosis of anti-NMDA encephalitis. Abbreviations: SI = signal intensity; WI = weighted imaging.

MCT is also associated with specific conditions, such as growing teratoma syndrome, autoimmune haemolytic anaemia, and immune encephalitis (Figure 7). Ovarian teratomas are implicated in up to 36% of patients presenting with anti-N-methyl-D-aspartate (NMDA) receptor encephalitis.^53^^,^^54^

Struma ovarii is a rare subtype of monodermal teratoma predominantly or entirely composed of thyroid tissue, accounting for approximately 3% of all mature teratomas.^55^ Unlike other mature teratomas, it typically occurs in women over the age of 40 and may be associated with hyperthyroidism.^44^^,^^51^^,^^56^ Malignant transformation is rare and most often manifests as papillary thyroid carcinoma.^51^

**Figure 8.**
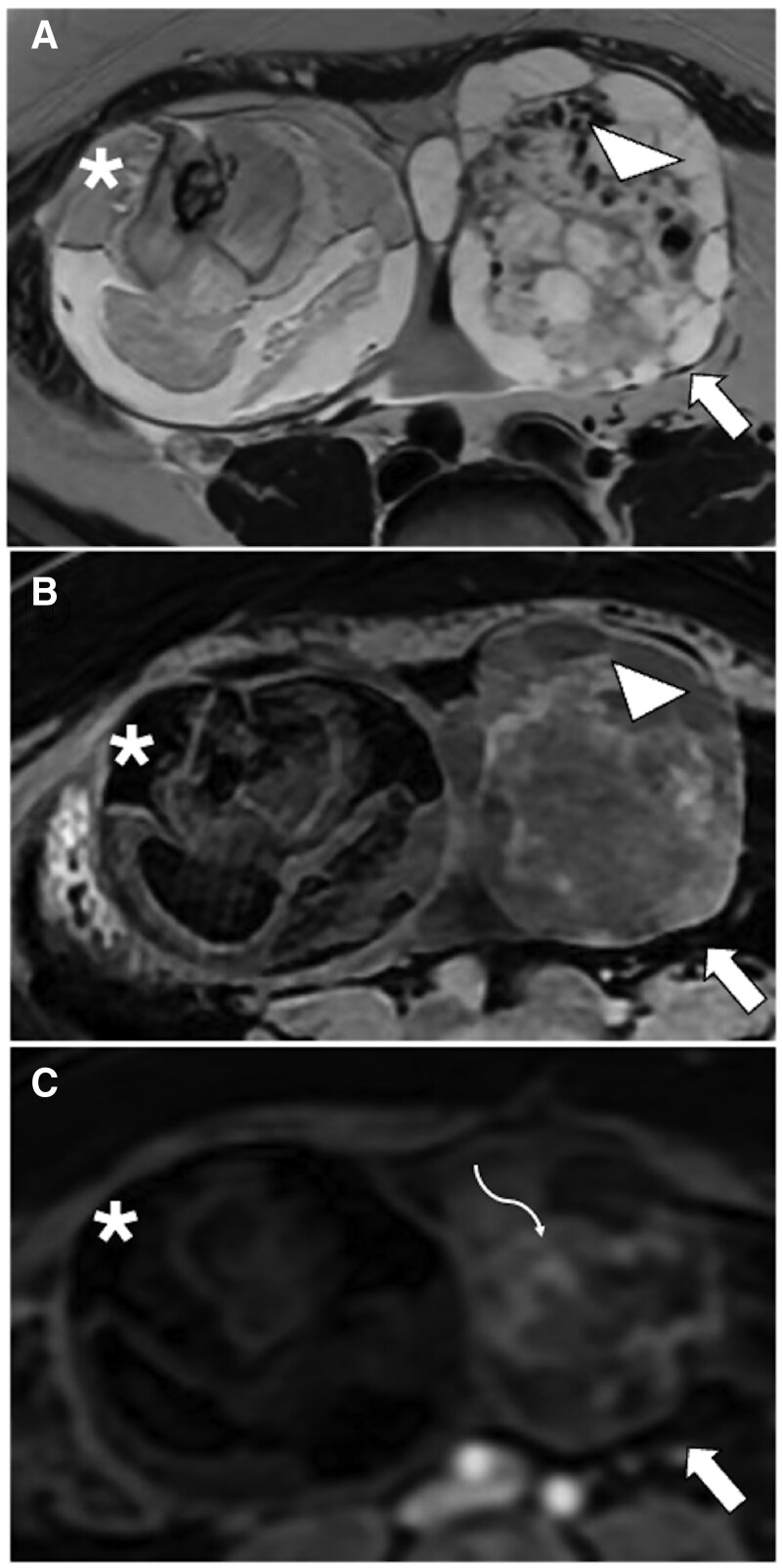
Struma ovarii. Struma ovarii typically presents as large multilocular lesion with loculi of variable SI on MRI. Some of the loculi (arrowheads in A and B) show low SI on T2-WI (A) and high SI on T1-WI (B) due to the colloid component. Vivid enhancement of the solid component is also visible (curved arrow) in the postcontrast T1-WI (C). A mature cystic teratoma can be seen on the right side of the struma ovarii (asterisk). Abbreviations: SI = signal intensity; WI = weighted imaging.

On MRI, struma ovarii commonly appears as a large multicystic mass with loculi of varying SI on T1- and T2-WI, and solid components. Due to the colloid component, some cystic spaces exhibit very low SI on T2-WI and corresponding variable SI on T1-WI.^44^^,^^51^^,^^53^^,^^57^^,^^58^ The solid component may demonstrate variable diffusion restriction and vivid postcontrast enhancement.^44^ Fat is usually absent within the lesion, but punctate foci of high SI on T1-WI, representing focal areas of viscous material or haemorrhage, are a common finding^55^^,^^57^^,^^58^ (Figure 8).

Immature teratoma is the second most common malignant germ cell tumour after dysgerminoma, accounting for less than 1% of ovarian cancers. It typically affects young patients, with a median age of 19 years, and is usually unilateral. However, a contralateral MCT is found in up to 20% of cases.^59^

On MRI, it presents as a solid-cystic lesion with irregular solid components, usually forming obtuse angles with the lesion walls, showing tiny cystic foci within it. Fat is typically present as scattered foci within the solid component rather than in the cystic cavity. As seen in MCTs, calcifications also exhibit a distinct pattern, appearing irregularly distributed rather than coarsened or tooth-like.^51^^,^^60^ Raised levels of alpha-fetoprotein may aid the diagnosis.^61^

### Dysgerminoma

Dysgerminoma is the most common malignant germ cell tumour, accounting for 1% to 2% of ovarian cancers. They primarily occur in adolescents and young adults or are diagnosed during pregnancy.^62^ The prognosis is excellent, with a 5-year overall survival rate approaching 100%.^63^

**Figure 9.**
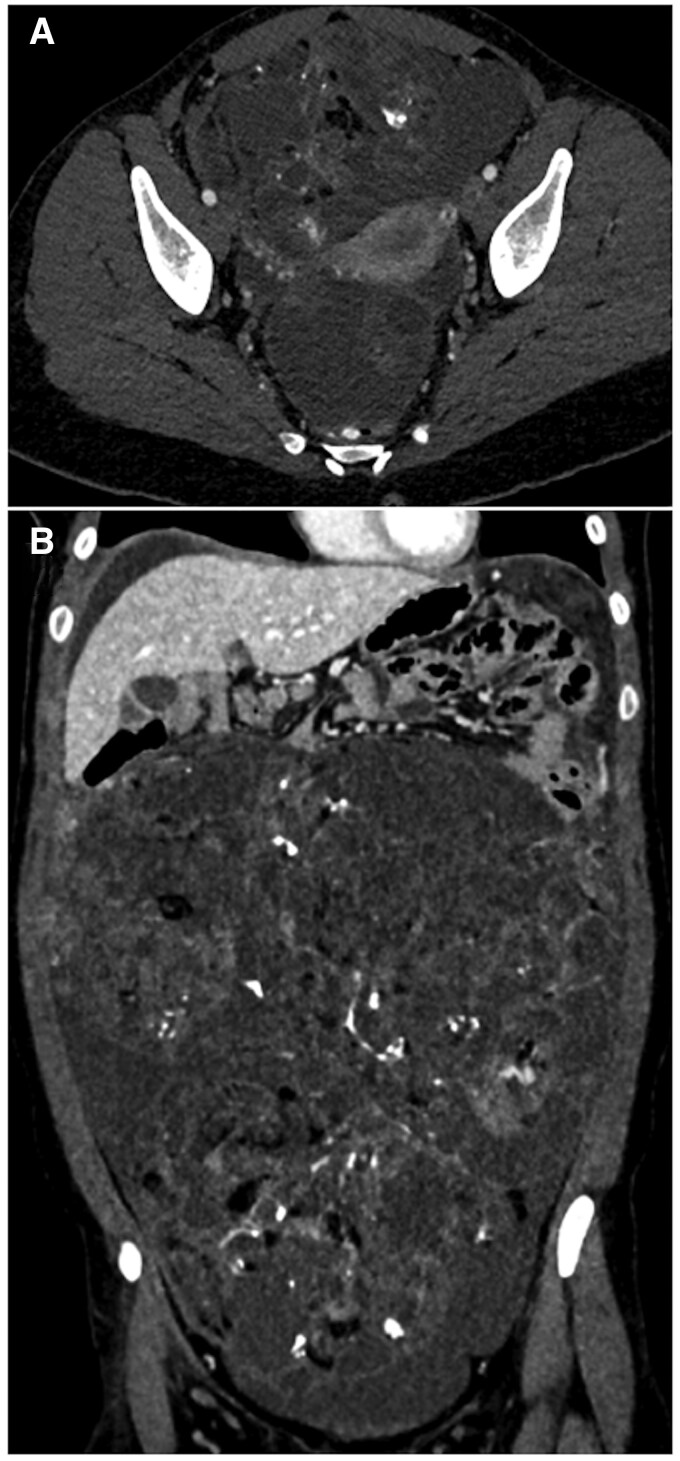
Dysgerminoma. CT images of a 19-year-old girl showing a large multilocular lesion with enhancing fibrovascular septa of different thicknesses, in keeping with ovarian dysgerminoma. Note the absence of peritoneal or nodal involvement despite the lesion size.

Dysgerminomas typically appear as large solid masses with nodular borders and variably thickened fibrovascular septa on ultrasound, CT, and MRI. Areas of haemorrhage or necrosis may be present within the lesion. These tumours are often diagnosed at an early stage, as they tend to spread late, primarily via the lymphatic system. Peritoneal carcinomatosis is rare and usually results from tumour rupture. Elevated serum lactate dehydrogenase level is a valuable finding for diagnosis and follow-up^62–64^ (Figure 9).

The primary differential diagnoses include other solid-cystic ovarian tumours such as juvenile granulosa cell tumours, which typically exhibit a “spongelike” appearance on imaging and are associated with symptoms related to oestrogen production (e.g. precocious puberty), choriocarcinomas and yolk sac tumours, which are associated with raised level of alpha-fetoprotein, and sclerosing stromal tumours, that can be distinguished by their characteristic centripetal enhancement pattern on imaging.^51^^,^^62^

## Tumours occurring at different sites

Some tumours, such as neuroendocrine tumours (NETs) or lymphoma, originate from cells that are not organ-specific and can consequently be found at multiple sites of the gynaecological tract and in different body districts.

### Neuroendocrine tumours

NETs of the gynaecological tract have recently been reclassified into low-grade (previously termed carcinoid) and high-grade subtypes (formerly known as small-cell and large-cell NET). The term “carcinoid” remains in use exclusively for ovarian carcinoids, which are associated with indolent behaviour and favourable prognosis. Ovarian carcinoids typically arise within MCTs and remain confined to the ovary.^65^^,^^66^

The cervix is the most affected site in the gynaecological tract, predominantly by high-grade tumours, and NETs account for 1%-6% of cervical cancers.^67^^,^^68^

**Figure 10.**
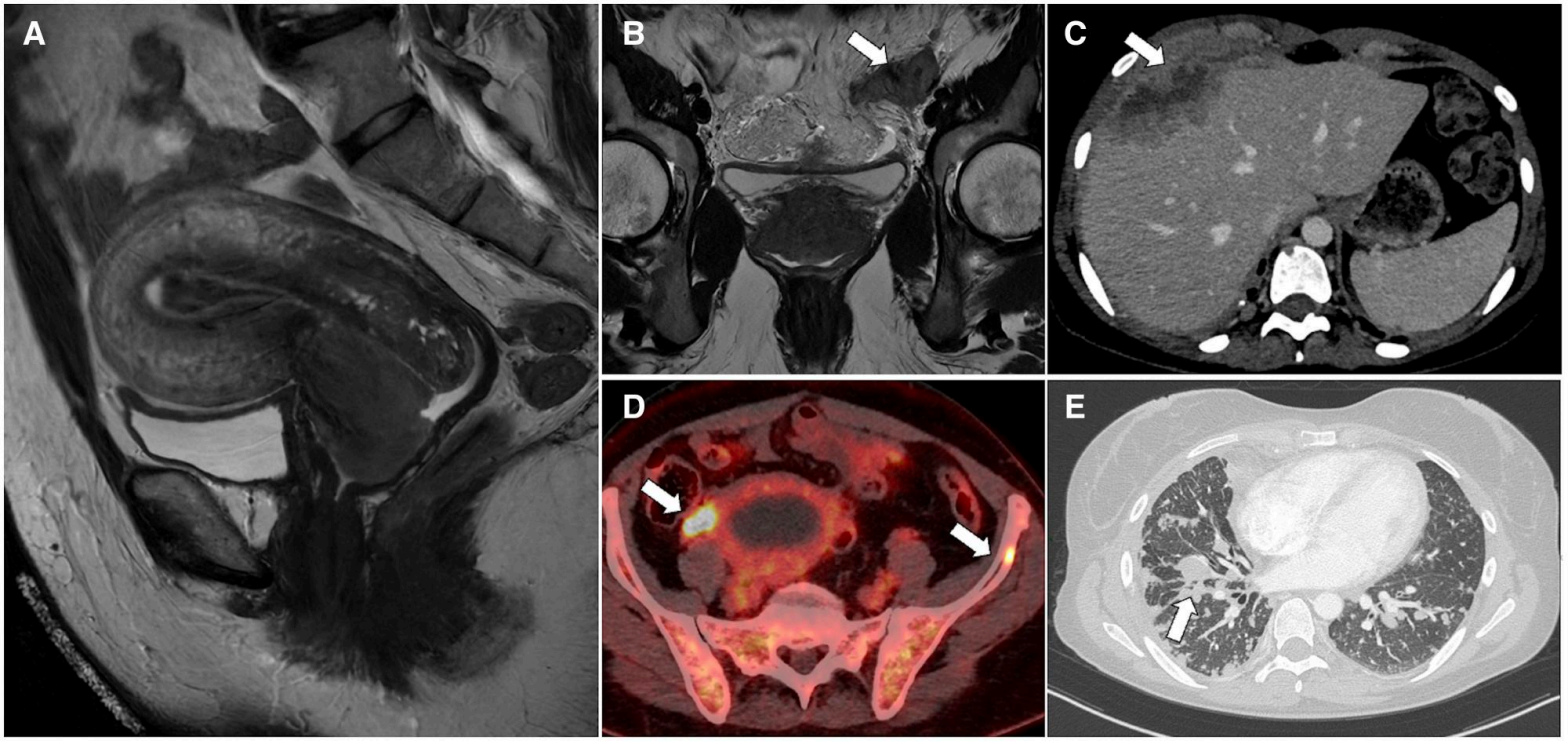
Neuroendocrine tumour of the cervix. Sagittal T2-WI (A) shows an intermediate-SI lesion in the anterior part of the cervix, which on MRI cannot be distinguished from a common squamous cell carcinoma. Due to the aggressive behaviour of neuroendocrine tumours, additional disease sites can be present (arrows), such as the peritoneum (B, C), ovaries (D), bones (D), and lungs (E). Abbreviations: SI = signal intensity; WI = weighted imaging.

Radiologists must be aware of the poor prognosis associated with neuroendocrine cervical cancer, which has a median survival of 21-22 months compared with approximately 10 years for SCC. This is due to its aggressive behaviour, characterized by higher rates of lymphovascular space invasion, nodal and distant metastases, and recurrent disease. Comprehensive assessment for distant metastases is essential when evaluating scans of patients with NEC^66^^,^^67^ (Figure 10). Notably, up to 26% of recurrences involve the brain, making brain imaging imperative in cases of suspected recurrence.^67^

While studies have shown that NETs have significantly lower mean ADC values (<0.90 × 10^−^³ mm^2^/s) than AC and SCC, their imaging features are generally nonspecific.^68^

### Lymphoma

The genitourinary system accounts for 3% of extra-nodal lymphomas, with diffuse large B-cell lymphoma being the most common subtype. Secondary involvement of gynaecological organs occurs 10× more frequently than primary lymphomas and is associated with a worse prognosis. Thus, assessment of other sites of disease is crucial. The ovaries are the most affected site, followed by the cervix, uterine corpus, and vagina.^69^ The peak age for uterine lymphomas is 40-50 years, while ovarian lymphomas have a broader age range (26-81 years), with a median age of 46 years.^70^

Lymphomas usually present as large, homogeneous masses, often exceeding 5 cm in size. Despite their size, areas of haemorrhage or necrosis are rare, and ascites is uncommon. Growth patterns can be nodular or diffuse. Nodular lymphomas appear as solid, round, well-defined masses with nodular borders, causing less mass effect than expected for their volume. In contrast, diffuse lymphomas lead to symmetrical enlargement of the affected organ, resulting in organomegaly while preserving the organ’s structure.^71^

**Figure 11.**
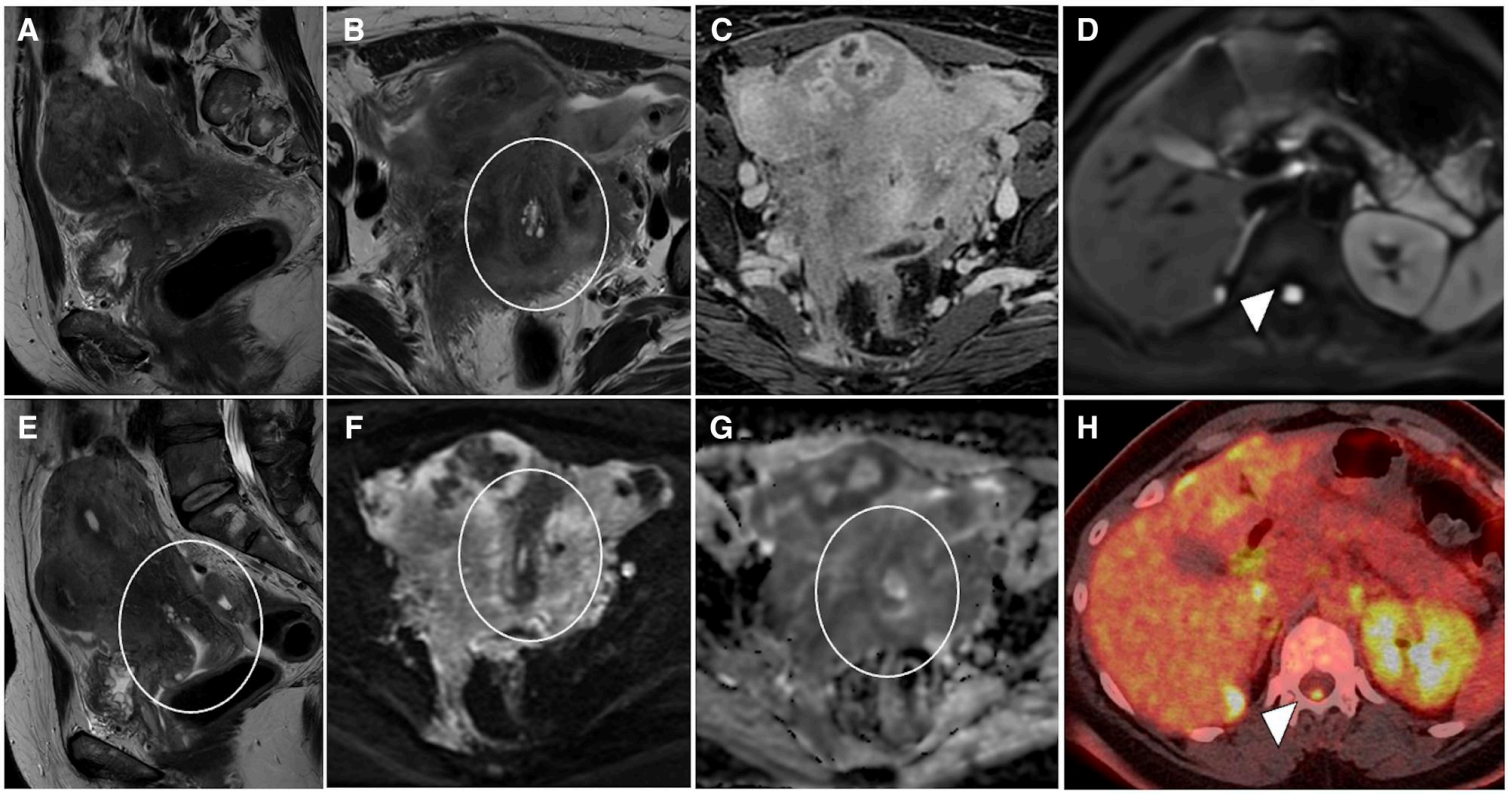
Diffuse large B-cell cervical lymphoma. On MRI, symmetrical and diffuse uterus enlargement can be noted due to lymphomatous infiltration. Despite the lesion size, no area of haemorrhage or necrosis is visible on postcontrast imaging (C). Preservation of the cervical mucosal layer (white circle), which is a distinguishing feature, is seen on axial and sagittal T2-WI (B, E), DWI images (F) and ADC map (G). Careful evaluation of additional disease sites (arrowheads) is needed, as seen on upper abdomen DWI (D) and FDG-PET/CT images (H). Abbreviation: WI = weighted imaging.

For example, cervical lymphomas typically manifest as large lesions with endophytic growth, causing diffuse enlargement of the cervix. A distinctive feature is the preservation of the cervical mucosa, which leads to missed detection during routine cervical cancer screenings^72^^,^^73^ (Figure 11).

Similarly, ovarian lymphomas are large masses that preserve the ovarian structure with normal follicles, often visible at the periphery. On MRI, they show homogeneous high SI on T2-WI, which is higher than that of other solid tumours and mild enhancement.^70^

Suspecting ovarian lymphoma is critical to avoid unnecessary surgical interventions, as the treatment choices are chemotherapy and radiotherapy, while surgery does not improve prognosis in these cases.^69^

### Aggressive angiomyxoma

Aggressive angiomyxoma is a rare, slow-growing mesenchymal tumour that typically affects the pelvis and perineum in middle-aged women. Vulva is the most common site (58%), followed by the vagina, buttocks, and cervix. Although these tumours lack metastatic potential, they exhibit an infiltrative growth and a high local recurrence rate (28%-72%), which can occur up to 15 years after initial diagnosis.^74^^,^^75^

Clinical examination often underestimates the extent of these tumours, making MRI crucial for identifying their mesenchymal origin and precisely defining their anatomical extent. This infiltrative growth tends to displace rather than invade adjacent structures. Translevator extension is a distinctive feature and aids in diagnosis.^3^^,^^76^^,^^77^ Surgery with negative margins is the preferred treatment, but achieving this can be challenging due to the tumour’s highly infiltrative pattern. MRI helps evaluate the involvement of surrounding structures such as organs, bones, vessels, and nerves, thereby crucial for surgical planning.

**Figure 12.**
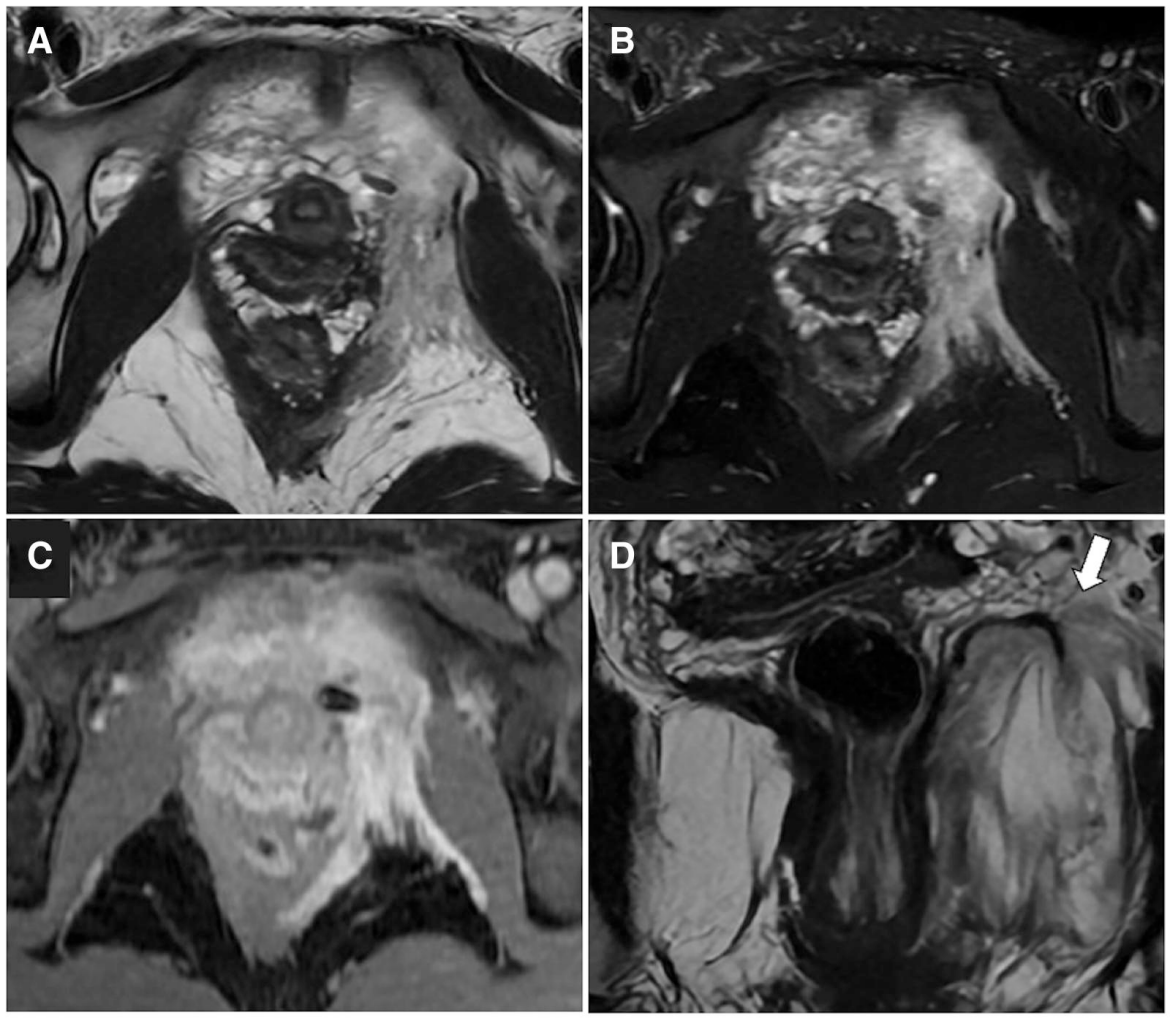
Aggressive angiomyxoma. MRI shows a solid mass with laminated appearance centred in the left pelvis, hyperintense on T2-WI (A). The lesion is more prominent on fat-saturated images (B) and shows vivid contrast enhancement (C). The characteristic translevator extension is visible on coronal images (arrow in D). Abbreviation: WI = weighted imaging.

On MRI, aggressive angiomyxomas manifest as solid multicompartmental masses with a laminated appearance. They show high SI on T2-WI due to their myxoid component, high water content, and vivid post-contrast enhancement but typically lack significant diffusion restriction (Figure 12). Additional features may include large collateral vessels, finger-like extensions, cystic changes, and occasional calcifications (6%-44%).^77^ In some cases, treatment with gonadotropin-releasing hormone agonists can reduce tumour size, reflected by decreased SI on T2-WI and reduced contrast enhancement on MRI.^3^

### Metastases

The ovary is the most commonly involved gynaecological organ in metastatic disease, followed by the fallopian tubes, while uterine involvement is rare.^76^^,^^78^ Ovarian metastases primarily originate from gastrointestinal cancers—particularly of the colon and stomach—followed by breast cancer, with lung and other malignancies being less frequent sources.^51^^,^^76^

Krukenberg tumours are a specific type of ovarian metastasis, characterized by the presence of signet ring cells, typically originating from the gastrointestinal tract, most commonly the stomach.^76^^,^^79^ Affected patients are generally younger compared with those with primary ovarian cancer.^76^

Distinguishing primary from secondary ovarian neoplasms can be challenging. On MRI, metastatic lesions exhibit variable SI on T2-weighted images depending on their composition—ranging from intermediate SI in solid lesions to high SI in cystic lesions. Metastases from breast and gastric cancers usually appear as predominantly solid, avidly enhancing masses, whereas metastases from other gastrointestinal sites or the biliary tract often present with cystic components that may mimic primary mucinous tumours.^51^^,^^79^ Imaging features favouring metastatic disease include bilateral ovarian involvement, a maximum lesion diameter of less than 13 cm, and the presence of peritoneal carcinomatosis.^51^

Metastatic involvement of the uterus is exceedingly rare and typically arises from breast or colorectal cancer. The cervix is almost invariably affected. Uterine metastases may present as either mass-forming lesions, predominantly in the cervix, or as diffuse infiltration leading to loss of the normal zonal anatomy and organ enlargement while preserving the overall uterine shape.^76^^,^^80^

Main imaging findings of the discussed tumours are summarized in Table 1.

**Table 1.** Summary

			Key findings
Uterine corpus			
	Leiomyosarcoma	Malignant	Only 0.2 % arise from a previous leyomioma Large myometrial-based lesion, irregular borders, intermediate-to-high SI on T2-WI, heterogeneous contrast enhancement for haemorrhage and necrosis The lesion shows higher SI than the endometrium or nodes on DWI and low-SI on ADC map
	Endometrial stromal sarcoma	Malignant	Endometrial-based lesion with significant myometrial invasion Intralesional hypointense areas on T2- and T1-WI representing preserved myometrium (“worm-like appearance”). Intramyometrial nodules, intravascular extension, and continuous spread to the surrounding structures via the fallopian tube or uterine ligaments are common
	Carcinosarcoma	Malignant	Large lesion within the endometrial cavity extending through the internal os Progressive, persistent enhancement may help distinguish it from other endometrial cancer subtypes High propensity for peritoneal involvement, nodal and distant metastases
Cervix			
	Gastric-type adenocarcinoma	Malignant	Solid-cystic morphology with small cysts within the solid component Infiltrative, endophytic growth centred within the cervical stroma leading to underestimation of the disease extension on MRI The adnexa and the peritoneum are common disease sites
Ovaries			
	Cystoadenofibroma	Predominantly benign, borderline and malignant forms are rare	Uni- or multilocular lesion with solid components showing markedly low SI on T2-WI and mild and progressive contrast enhancement. Three morphological patterns: black-sponge, carpet-like, or purely cystic Common mimicker of other epithelial tumours
	Lipid poor teratoma	Benign. Malignant transformation is possible	15% of mature cystic teratomas No intracavital fat in the cystic cavity but fat can be found within the cystic wall or Rokitansky nodule
	Struma ovarii	Benign. Malignant transformation is possible	Women >40 years old. Association with hyperthyroidism described Large multilocular lesion. Loculi with different SI, some of them showing very low SI on T1-WI and variable SI on T2-WI, due to colloid component Usually, absence of fat
	Immature teratoma	Malignant	Young patients Large irregular solid component and scattered fatty foci within the solid component rather than in the cystic cavity D.d.: mature cystic teratoma Raised level of alpha-fetoprotein
	Dysgerminoma	Malignant	Adolescents and young adults Large solid masses with nodular borders and variably thickened, enhancing fibrovascular septa Serum lactate dehydrogenase for diagnosis and follow-up
Multiple sites			
	Neuroendocrine tumours	Malignant	No specific imaging features Ovarian carcinoid: usually in association with MCT; indolent behaviour and favourable prognosis Cervical cancer: poor prognosis due to high rate of nodal and distant metastases and of recurrent disease
	Lymphoma	Malignant	Secondary involvement of gynaecological organs is more frequent than primary lymphoma Large, homogeneous mass. Areas of haemorrhage or necrosis are rare Nodular pattern: sharply demarcated round solid lesion with nodular borders causing less mass effect than expected for tumour size Diffuse pattern: diffuse and symmetrical enlargement of the involved organ with preservation of the organ structure (eg, preservation of cervical mucosal layer or normal follicles at ovarian periphery)
	Aggressive angiomyxoma	Benign but locally invasive	Typically found in pelvis and perineum of middle-aged women High rate of local recurrence due to infiltrative involvement of surrounding structures Solid multicompartmental mass with laminated appearance, showing high SI on T2-WI and vivid post-contrast enhancement Trans-levator ani muscle extension is typical
	Metastases	Malignant	Gastrointestinal and breast cancer are the most common primary malignancies Ovarian metastases can be solid or mixed solid-cystic lesions (mimicking primary mucinous neoplasms) Uterine metastases present as mass-forming lesions (cervix) or diffuse uterine enlargement with loss of the zonal anatomy and preserved organ shape

## Conclusions

The management of rare tumours is raising significant awareness as unmet clinical needs due to the lack of specific treatment options. Radiologists can help address this challenge by understanding the clinical behaviour and imaging correlates that can aid in accurate diagnosis. Indeed, assessing tumour characteristics and disease extent can support clinical decision-making and treatment planning. Active participation in multidisciplinary team discussions is equally important, as it allows radiologists to gain up-to-date insight into clinical needs, ultimately improving patients’ management in these challenging scenarios.
